# Working alliance and adherence mediate the effect of guidance in a web-based program for participants with mild to moderate depressive symptoms: A secondary mediation analysis

**DOI:** 10.1016/j.invent.2022.100593

**Published:** 2022-11-26

**Authors:** Oliver Thomas Bur, Laura Luisa Bielinski, Samantha Krauss, Andrea Häfliger, Jasmin Guggisberg, Tobias Krieger, Thomas Berger

**Affiliations:** a Department of Clinical Psychology and Psychotherapy, University of Bern, Bern, Switzerland; b Department of Developmental Psychology, University of Bern, Bern, Switzerland

**Keywords:** Web-based self-help program, Internet intervention, Depression, Guidance, Working alliance, Factorial trial, Problem-solving therapy, Mediation, Adherence

## Abstract

Guided web-based self-help programs for individuals with depressive symptoms have shown to be more efficacious than unguided programs. However, research has paid little attention to why guided interventions are superior. The present study investigated whether working alliance and adherence to the program mediated the effect of guidance on depressive symptom outcome.

The study is a secondary analysis of a randomized factorial trial. In the trial, 302 adults with mild to moderate depressive symptoms (Patient Health Questionnaire–9 score: 5–14) were randomized to either a guided or an unguided group. All participants received access to a web-based self-help program based on problem-solving therapy. Working alliance with the treatment providers was assessed using an adapted version of the Working Alliance Inventory for Guided Internet Interventions two weeks (early-treatment) and eight weeks (post-treatment) after pre-treatment. The primary outcome was depressive symptoms at post-treatment.

The total working alliance score was significantly higher for guided participants compared to unguided participants (at early-treatment: *t*_248.6_ = −3.36, *p* < .001, *d* = 0.42, at post-treatment: *t*_194.9_ = −4.77, *p* < .001, *d* = 0.66). The total working alliance score correlated significantly with the change in depressive symptoms for guided (*rs* = 0.16, 0.34) and unguided participants (*rs* = 0.26, 0.23). The WAI-I total score statistically mediated the relationship between guidance and outcome (at early-treatment: *B* = −0.028, at post-treatment: *B* = −0.053). Furthermore, the subscale tasks (at post-treatment: *B* = −0.051), the subscale goals (at early-treatment: *B =* −0.031 and at post-treatment: *B* = −0.052), and adherence to the program (*B* = −0.034) mediated the relationship between guidance and outcome. Finally, in a multiple mediation model both early-treatment working alliance and adherence to the program (*B* = −0.050) mediated the relationship between guidance and outcome.

These findings indicate that guidance increases working alliance to treatment providers as early as two weeks after treatment beginning. The alliance predicts outcome and mediates the relationship between guidance and outcome. Participants' agreement with tasks and goals of a program seems to be more important than the bond with treatment providers. Treatment providers might therefore attune web-based programs to the preferences and expectations of participants. In addition to the working alliance, adherence to the program co-mediates the relationship between guidance and outcome.

## Highlights

• Guided participants showed a higher working alliance with treatment providers compared to unguided participants
• Working alliance at early- and post-treatment was associated with change in depressive symptoms
• Working alliance and adherence to the program mediated the relationship between guidance and depressive symptom outcome
• Participants’ agreement with tasks and goals of a program seems to be more important than the bond with treatment providers

## 1 Introduction

Guided web-based programs have shown to reduce depressive symptoms efficaciously. They are usually associated with larger effects than unguided web-based programs and tend to achieve equivalent effects to face-to-face psychotherapies (Andersson et al., 2014; Bur et al., 2022; Carlbring et al., 2018; Karyotaki et al., 2021; Moshe et al., 2021). While there has been increased attention to whether guided interventions are superior to unguided interventions, research has paid little attention to *why* guidance is associated with better treatment outcomes. Consequently, we do not know how guidance leads to greater symptom improvement. However, by understanding the processes that explain the effect of guidance, one might identify and convey the essential facets of guidance and understand what is needed to improve treatment outcomes.

One approach to investigate how guidance works is to examine possible mediators through which guidance might achieve its effect. A mediator statistically accounts for a relationship between an independent and a dependent variable (Kazdin, 2007; Kazdin, 2009). This can imply that the mediator itself is the mechanism that explains change precisely. More likely, however, a mediator serves as a proxy for one or more variables with which it correlates (Kazdin, 2009). In that case, the mediator points at the underlying mechanism that produces change and is, therefore, a first hint of how change occurs.

An extensively studied process variable and potential mediator of symptom change in face-to-face psychotherapy is the working alliance. This concept traces back to Bordin (1979), who defines the working alliance as 1) the emotional bond between a patient and a therapist, 2) shared agreement with the tasks of therapy, and 3) shared agreement with the goals of therapy. The working alliance is often measured with the Working Alliance Inventory (WAI; Horvath and Greenberg, 1989), and many studies underpin that a good working alliance is associated with a better treatment outcome. Several meta-analyses have shown that the alliance predicts treatment outcome in face-to-face therapies (*rs* = 0.22–0.28; Flückiger et al., 2018; Horvath et al., 2011; Martin et al., 2000). Furthermore, a recent review has shown that in most studies, depressive symptom change was partially mediated by the alliance (Baier et al., 2020).

The importance of the working alliance seems not to be restricted to face-to-face psychotherapy. Reviews on the alliance in online therapy concluded that independent of communication modalities (e.g., email, videoconferencing), diagnostic groups, and amount of contact between clients and therapists, client-rated alliance scores were high, and roughly equivalent to alliance ratings found in studies on face-to-face psychotherapy (Berger, 2017; Pihlaja et al., 2018). Furthermore, several meta-analyses have shown that the working alliance is associated with better outcomes in guided web-based programs (*rs* = 0.20–0.28; Flückiger et al., 2018; Kaiser et al., 2021; Probst et al., 2019). These findings are noteworthy because in guided self-help interventions, the therapists' tasks are often limited to reinforce participants' independent work, provide feedback on participants' progress, and answer participants' questions.

The working alliance's role in guided web-based programs for depression has not been conclusively clarified. Within the three meta-analyses on web-based programs mentioned in the previous paragraph, eight studies investigated depressive symptoms as the primary outcome. Whereas half of the studies reported significant positive correlations between the working alliance and depressive symptom change (Anderson et al., 2018; Meyer et al., 2015; Gómez Penedo et al., 2020; Preschl et al., 2011), half did not (Andersson et al., 2012; Hadjistavropoulos et al., 2017; Ormrod et al., 2010; Richards et al., 2013). Furthermore, Preschl et al. (2011) found the positive association only for WAI measures assessed at post- but not mid-treatment. Thus, it is unclear whether the alliance promoted depressive symptom reduction, whether patients with fewer depressive symptoms rated the working alliance as higher, or whether a third variable influenced both the alliance ratings and depressive symptoms. Finally, the eight studies used different measures to assess the working alliance and provided varying amounts of guidance during treatment. This heterogeneity complicates conclusions about the relationship between the working alliance and depressive symptoms. Consequently, more studies are needed that clarify the relationship of early working alliance ratings and depressive symptoms in guided web-based programs.

Apart from the working alliance, adherence, i.e., the extent to which participants use a self-help program, could also mediate the effect of guidance on the outcome. On the one hand, some studies have found that guidance is associated with higher adherence (Baumeister et al., 2014; Bur et al., 2022; Donkin et al., 2011; Karyotaki et al., 2021). On the other hand, several studies found an association between adherence and treatment outcome (Donkin et al., 2011; El Alaoui et al., 2016; Fuhr et al., 2018; Karyotaki et al., 2017; Newby et al., 2014). Consequently, guidance may encourage participants to use the program more intensively, leading to better outcomes. It should be noted, however, that not all studies find an association between adherence and outcome (e.g., Farrer et al., 2014; Donkin et al., 2013).

The current study is a secondary analysis of data from a randomized factorial trial. In the trial, we investigated the context of support of a web-based self-help program for depressive symptoms (Bur et al., 2022). We found that guidance was associated with significantly better outcomes at post-treatment. Although several meta-analyses have suggested this finding before (Karyotaki et al., 2021; Moshe et al., 2021; Spek et al., 2007), to the best of our knowledge, no study has so far investigated why guided interventions seem to be superior to unguided interventions.

Since several meta-analyses have found that working alliance is associated with better outcomes in internet-based treatments (Flückiger et al., 2018; Kaiser et al., 2021; Probst et al., 2019), we hypothesized that the working alliance might play an important role in explaining the superiority of guided programs. Therefore, we investigated three research questions: First, we investigated whether guided participants showed a higher working alliance with the treatment providers than unguided participants. Second, we investigated whether the working alliance correlated positively with depression change scores. Third, we took a closer look at the role of the working alliance as a possible mediator for the relationship between guidance and outcome. We hypothesized that similar to most face-to-face psychotherapy studies (cf. Baier et al., 2020), the working alliance mediates the effect of guidance on depressive symptom outcomes. Furthermore, we investigated whether adherence also plays a role in explaining the superiority of guided programs.

## 2 Methods

### 2.1 Participants

Participant data for the present analyses came from the HERMES trial (Bur et al., 2022). Individuals were allowed to take part in the study if they 1) were at least 18 years of age, 2) indicated mild to moderate depressive symptoms on the Patient Health Questionnaire-9 (PHQ-9 score between 5 and 14; Kroenke et al., 2001), 3) provided written informed consent, 4) had access to the internet and an email account, and 5) provided an emergency contact. Individuals were not allowed to take part in the study if they 1) reported a present or past psychotic or bipolar disorder, or 2) indicated increased suicidal tendencies on the Suicidal Behavior Questionnaire-Revised (SBQ-*R* > 7; Osman et al., 2001). Participants were recruited online via our study website. Participants had to complete and return a consent form before completing the pre-treatment online questionnaire, which checked for eligibility. Of note, participants taking medication or seeing a psychotherapist could participate in the study. Participants were not compensated for taking part in the study.

Participant characteristics are shown in Table 1. Participants of this study were on average 38.4 years of age (*SD* = 13.7, range: 19–78). Most participants were female (72.8 %), single (60.9 %) and Swiss (50.7 %) or German (43.7 %). Furthermore, most participants reported a university degree (58.9 %) and part- or full-time employment (59.0 %). About one-third of the participants were in concurrent psychological treatment (30.8 %), and about one-fifth used prescribed medication for mental disorders (21.2 %) at pre-treatment.

**Table 1:** Pre-treatment demographics and characteristics overall, for guided, and for unguided groups.

Characteristic							
	Total sample (N = 302)		Guided (n = 150)		Unguided (n = 152)		Statistic
	n	%	n	%	n	%	
Age							
Mean (SD)	38.4 (13.7)		38.1 (13.2)		38.8 (14.2)		
Range	19–78		19–69		19–78		*t*_298.9_ = 0.45, *p* = .65
Gender							
Male	81	26.8	37	24.7	44	28.9	
Female	220	72.8	113	75.3	107	70.4	
Non-binary	1	0.4	–	–	1	0.7	*χ*^2^_2_ = 1.76, *p* = .42
Origin of birth							
Switzerland	153	50.7	73	48.7	80	52.6	
Germany	132	43.7	70	46.7	62	40.8	
Other	17	5.6	7	5.6	10	6.6	*χ*^2^_3_ = 1.11, *p* = .77
Marital Status							
Single	184	60.9	98	65.3	86	56.6	
Married	89	29.5	36	24.0	53	34.9	
Divorced/widowed	24	8.0	13	8.7	11	7.2	
Other	5	1.6	3	2.0	2	1.3	*χ*^2^_3_ = 4.38, *p* = .22
Education							
Less than high school	5	1.7	3	2.0	2	1.3	
High school diploma	60	19.9	25	16.7	35	23.0	
University	178	58.9	91	60.7	87	57.2	
Apprenticeship	59	19.5	31	20.7	28	18.4	*χ*^2^_2_ = 2.06, *p* = .36
Employment							
Full-time paid work	66	21.9	37	24.7	29	19.1	
Part-time paid work	115	38.1	52	34.7	63	41.4	
Unemployed	20	6.6	9	6.0	11	7.2	
Student	80	26.5	40	26.7	40	26.3	
At-home parent	5	1.6	4	2.7	1	0.7	
Retired	16	5.3	8	5.3	8	5.3	*χ*^2^_5_ = 4.01, *p* = .55
Current psychological treatment	93	30.8	47	31.3	46	30.3	*χ*^2^_1_ = 0.04, *p* = .84
Current medication	64	21.2	29	19.3	35	23.0	*χ*^2^_1_ = 0.61, *p* = .43

### 2.2 Study design

HERMES was a randomized full factorial trial, which included four experimental factors (1; guidance, 2; a diagnostic interview, 3; a motivational interviewing module, 4; automated emails). Each factor was varied at two levels (either present, coded as +1, or absent, coded as −1; i.e., effect coded), resulting in a 16-condition (2 × 2 × 2 × 2) trial (Bur et al., 2022). In the present paper, we focused on comparing guided vs. unguided conditions since guidance was the only factor that significantly improved outcomes. The ethics committee of the canton of Bern approved the study on January 20, 2020 (2019-01795), and the study is registered at ClinicalTrials.gov (NCT04318236).

### 2.3 Procedure

HERMES participants were randomized by Qualtrics (XM) to either guided or unguided conditions. The randomization was stratified for mild (PHQ-9: 5–9) or moderate (PHQ-9: 10–14; Kroenke et al., 2001) depressive symptoms, and the randomization schemes were concealed from both the participants and the study staff. During the eight weeks of working on the web-based program, guided participants (*n* = 150) were supported by clinical psychologists (supervised master students in their last term of a graduate program in clinical psychology and psychotherapy and a Ph.D. student in clinical psychology and psychotherapy). At the beginning of the treatment, the psychologists introduced themselves and explained that the participant could ask questions at any time. The psychologists wrote an email to the participants each week, to provide feedback on the participants' behaviour and progress in the self-help program. These emails were sent in a secured email system integrated into the self-help program. Emails did not include further therapeutic advice. The most important aspects of the feedback were crediting and reinforcing participants' independent work. The psychologists asked if participants were facing any problems and if they needed support, whenever guided participants did not work for a week with the program. Psychologists answered questions within the next three days. In total, the psychologists sent 1140 messages to the 139 participants who had logged in at least once (8.2 messages per participant). Furthermore, the psychologists spent 107 min per participant (*SD* = 62.8) and 12.6 min per message (*SD* = 6.5). Unguided participants received an automated introductory email. They had no further contact with the treatment providers, except if they asked technical questions regarding the use of the program (Bur et al., 2022).

### 2.4 The self-help program

All participants received full access to the 8-week web-based self-help program *HERMES*. The program is based on problem-solving therapy (PST; Nezu et al., 2012) developed at the University of Bern. It consists of a general introduction to the rationale of PST and three toolkits. The self-help program content is displayed through text, audio, and videos, including case examples and several exercises. The toolkits are organized around the subjects of feeling, thinking, and acting, which include several topics. Toolkit 1 (Feeling) deals with mindfulness, emotion observation and regulation, and relaxation. Toolkit 2 (Thinking) deals with self-criticism, cognitive restructuring, and healthy thinking. Toolkit 3 (Acting) deals with defining problems, thinking of, and choosing solutions, acting out a solution plan, and evaluating problem-solving attempts.

### 2.5 Measures

For the current study, we used assessments measured at pre-treatment, two weeks after pre-treatment (early-treatment), and eight weeks after pre-treatment (post-treatment). All assessments were self-reports and completed via Qualtrics. In the following paragraphs, the measures relevant to the analyses in this paper are discussed in detail. A full list of measures assessed in the trial can be found in a different publication (Bur et al., 2021).

### 2.6 Primary outcome

*Patient Health Questionnaire-9 (PHQ-9;*Kroenke et al., *2001**).* The primary outcome was the PHQ-9 at post-treatment. The PHQ-9 is a validated 9-item self-report measure of depressive symptoms. Each item can be answered from “0” (not at all) to “3” (nearly every day) resulting in a total range of 0 to 27 (Kroenke et al., 2001). Cronbach's *α* for post-intervention data was 0.84. Since the PHQ-9 served as an inclusion criterion, pre-treatment data were affected by substantial restriction of range and distorted reliability estimates (Stauffer and Mendoza, 2001).

### 2.7 Mediators

#### 2.7.1 Working Alliance

Working alliance was assessed using the Working Alliance Inventory for Guided Internet Interventions (WAI-I; Gómez Penedo et al., 2019). The WAI-I is a validated 12-item self-report measure for the working alliance. It consists of three subscales, i.e., tasks, goals, and bond. Each subscale consists of four items, which can be answered from “1” (rarely) to “5” (always), resulting in a total range of 12 to 60. We adapted the wording of the original WAI-I to fit the specifications of our study. Specifically, the four items of the bond subscale were rephrased to refer to the acceptance and trust between the patient and the treatment providers. The treatment providers included both the human contact prior to the treatment as well as the contact with psychologists who provided guidance. Therefore, the WAI-I questionnaire was answerable for both guided and unguided participants. In the original version, the items of the bond subscale referred to the acceptance and trust between the patient and the psychologist who provided guidance only (Gómez Penedo et al., 2019*).* The four items of the goals subscale and the four items of the tasks subscale remained the same as in the original WAI-I, i.e., they referred to the patient's agreement with the web-based program's goals and tasks. The WAI-I was assessed at early-treatment and at post-treatment. Cronbach's *α* at early-treatment was 0.90 for the total score, 0.87 for the subscale tasks, 0.81 for the subscale goals, and 0.88 for the subscale bond, respectively.

#### 2.7.2 Adherence

We defined adherence as the extent to which participants used the self-help program. Therefore, we calculated a composite score by averaging the *z*-scores of the following indicators: number of clicks, number of topics worked on, number of completed exercises, and time spent on the program. We calculated the adherence score for the time between pre- and post-treatment.

### 2.8 Statistical analyses

**Fig. 1:**
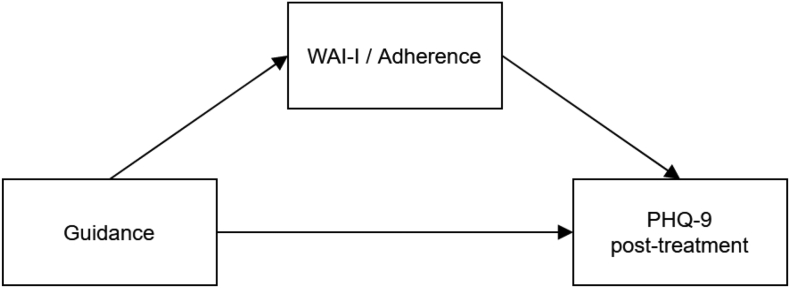
Single mediation models with working alliance or adherence as mediator *Note.* Pre-treatment depression scores were controlled for. PHQ-9: Patient Health Questionnaire-9; Primary Outcome WAI-I: Working Alliance Inventory for Guided Internet Intervention Adherence: Composite score of clicks, exercises, topics, and time spent on the self-help program. Adherence was calculated for the time between pre- and post-treatment.

**Fig. 2:**
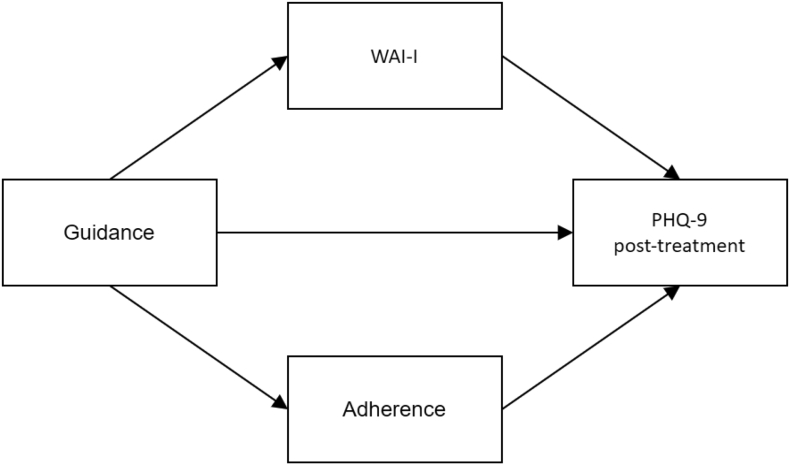
Multiple mediation model with working alliance and adherence as mediators *Note.* Pre-treatment depression scores were controlled for. PHQ-9: Patient Health Questionnaire-9; Primary Outcome WAI-I: Working Alliance Inventory for Guided Internet Intervention Adherence: Composite score of clicks, exercises, topics, and time spent on the self-help program. Adherence was calculated for the time between pre- and post-treatment.

We tested group differences between the unguided and guided conditions with *t*-tests for continuously distributed variables and χ^2^-tests of independence for categorical variables for pre-treatment and demographic measures. For the associations of the working alliance and depressive symptom outcome, we calculated partial correlations. Thereby, we correlated WAI-I measures with the pre- to post-treatment change in depressive symptoms while controlling for pre-treatment depressive symptoms prior to the allocation to the guidance conditions. For the relationship between adherence and working alliance and adherence and depressive symptoms, we calculated correlations with Kendall's τ. In the mediation analyses, we first tested in separate models whether the effect of guidance on depressive symptoms at post-treatment was mediated by 1) working alliance (at early- and post-treatment) and 2) adherence, while controlling for the level of pre-treatment depressive symptoms (Fig. 1). Finally, we tested a multiple mediation model adding both potential mediators in parallel to the model (Fig. 2). To test our mediation hypotheses, we employed structural equation modeling (SEM), using the *lavaan* package (Rosseel, 2012) for R (Version 3.5.2) and R Studio (Version 1.3.1093). To deal with missing values, we employed full information maximum likelihood estimation to fit models directly to the raw data (Schafer and Graham, 2002). Model fit was assessed with the comparative fit index (CFI), the standardized root mean square residual (SRMR), and the root mean square error of approximation (RMSEA). Good fit was indicated by values equal to or higher than 0.94 for CFI, equal to or <0.08 for SRMR, and equal to or <0.06 for RMSEA (Hu and Bentler, 1999). We used multiple indicators to measure working alliance and depressive symptoms as latent variables, which allowed us to control for measurement error. Working alliance was measured by three indicators (i.e., the three subscales of the WAI-I) and depressive symptoms were measured by three random parcels consisting of the items from the PHQ-9. To examine the significance of the indirect effects, we computed bootstrapped bias-corrected 95 % confidence intervals. Bootstrapping runs many data simulations based on randomly selected observations with replacements from the data. Therefore, it does not make assumptions regarding the shape of the distribution of the indirect effect but uses its empirical distribution. Bootstrapping is regarded as superior to the method of Baron and Kenny (1986) because it has greater statistical power and yields more accurate estimates of the confidence intervals (Shrout and Bolger, 2002). The point estimate of the indirect effect is considered statistically different from zero, if zero is not included in the 95 % confidence interval. The indirect mediation effect sizes were interpreted as 0.03 being a small effect, 0.07 being a medium effect, and 0.12 being a large effect.

## 3 Results

### 3.1 Pre-treatment evaluation and dropouts from the study

There were no pre-treatment group differences between the guided and the unguided group regarding demographics, depressive symptoms, current psychotherapeutic treatment, and current medication (Table 1). Participants who did not fill in post-treatment questionnaires were considered dropouts. Of the total sample size (*n* = 302), 208 individuals (68.9 %) completed post-treatment questionnaires. There were no significant differences in demographics for completers and dropouts (all *ps* > 0.05). However, guided participants were more likely to complete post-treatment questionnaires (*t*_1_ = 4.60, *p* = .03). Furthermore, participants with a higher working alliance rating at early-treatment (*t*_65.1_ = −2.14, *p* = .04) were more likely to complete post-treatment questionnaires. Little's MCAR test resulted in χ^2^ = 61.39 (df = 48, *p* > .05), indicating that data was missing at random.

### 3.2 Intervention outcomes

Results from the factorial trial have been published in a previous paper (Bur et al., 2022). Both guided (*d* = 0.72) and unguided participants (*d* = 0.38) showed a statistically significant decrease in depressive symptoms at post-treatment. There was a small but statistically significant between-group effect in favour of guidance (*d* = 0.15).

### 3.3 Working alliance

Results concerning the working alliance were not reported in the main outcome paper and are reported here. Descriptive information on means and standard deviations of depressive symptoms and working alliance across time is reported in Table 2. At early-treatment, the total score of the working alliance was significantly higher for guided participants compared to unguided participants (*t*_248.6_ = −3.36, *p* < .001, *d* = 0.42). For the two subscales tasks (*t*_248.1_ = −0.11, *p* = .92, *d* = 0.01) and goals (*t*_247.2_ = −1.74, *p* = .08, *d* = 0.22), there was no significant difference between the two groups. However, for the subscale bond, the score was significantly higher for guided participants (*t*_248.1_ = −5.64, *p* < .001, *d* = 0.71). A similar pattern emerged at post-treatment. The total score of the working alliance was significantly higher for guided participants compared to unguided participants (*t*_194.9_ = −4.77, *p* < .001, *d* = 0.66). For the subscale tasks there was no significant difference between both groups (*t*_202.5_ = −1.92, *p* = .06, *d* = 0.27). However, in the two subscales goals (*t*_198.5_ = −2.90, *p* < .01, *d* = 0.40) and bond (*t*_170.6_ = −5.88, *p* < .001, *d* = 0.84), the score was significantly higher for guided participants.

**Table 2:** Observed means and standard deviations of depressive symptoms (PHQ-9), working alliance (WAI-I), and adherence.

	Pre-treatment		Early-treatment		Post-treatment	
Measure	Guided (n = 150)	Unguided (n = 152)	Guided (n = 128)	Unguided (n = 127)	Guided (n = 111)	Unguided (n = 97)
	M (*SD*)	M (*SD*)	M (*SD*)	M (*SD*)	M (*SD*)	M (*SD*)
PHQ-9	9.43 (3.75)	8.97 (3.94)	8.18 (3.31)	8.13 (4.28)	6.71 (3.85)	7.73 (5.14)
WAI-I						
Total			3.27 (0.73)	2.96 (0.75)	3.62 (0.70)	3.14 (0.75)
Tasks			2.87 (0.86)	2.86 (0.80)	3.35 (0.91)	3.11 (0.85)
Goals			3.28 (0.75)	3.11 (0.81)	3.57 (0.76)	3.27 (0.76)
Bond			3.65 (1.03)	2.9 (1.09)	3.95 (0.92)	3.04 (1.24)
Adherence					0.85 (3.01)	−0.54 (2.04)

*Note.* Early-treatment = two weeks after treatment begin, post-treatment = eight weeks after treatment begin.

PHQ-9: Patient Health Questionnaire-9; Primary Outcome.

WAI-I: Working Alliance Inventory for Guided Internet Intervention; Mediator.

Adherence: Composite score of clicks, exercises, topics, and time spent on the self-help program. Adherence was calculated for the time between pre- and post-treatment.

Early-treatment = two weeks after treatment begim, p.

### 3.4 Working alliance, adherence and change in depressive symptoms

The total score of the working alliance and change in depressive symptoms correlated significantly at early- and post-treatment for the guided and unguided group (*rs* = 0.16–0.34). Further partial correlations, controlling for pre-treatment depressive symptoms, between WAI-I (sub)scales and change in depressive symptoms can be found in Table 3. The composite adherence score significantly correlated with the pre-to-post changes in PHQ-9 (Kendall's τ = 0.11, *p* = .025). Furthermore, adherence also correlated with early-treatment working alliance (Kendall's τ = 0.17, *p* < .01) and post-treatment working alliance (Kendall's τ = 0.26, *p* < .01).

**Table 3:** Partial correlations between change in depressive symptoms (PHQ-9) and the total score and subscales of the working alliance (WAI-I).

Early-treatment								
WAI-I	Total		Tasks		Goals		Bond	
	Guided	Unguided	Guided	Unguided	Guided	Unguided	Guided	Unguided
WAI-I								
Total								
Tasks	0.83***	0.82***						
Goals	0.87***	0.81***	0.78***	0.72***				
Bond	0.75***	0.81***	0.31**	0.40***	0.43***	0.40***		
PHQ-9-change	0.16*	0.26**	0.27**	0.22*	0.15	0.25*	0.00	0.19


Post-treatment								
WAI-I	Total		Tasks		Goals		Bond	
	Guided	Unguided	Guided	Unguided	Guided	Unguided	Guided	Unguided
Total								
Tasks	0.87⁎⁎⁎	0.78⁎⁎⁎						
Goals	0.86⁎⁎⁎	0.83⁎⁎⁎	0.78⁎⁎⁎	0.81⁎⁎⁎				
Bond	0.72⁎⁎⁎	0.75⁎⁎⁎	0.35⁎⁎⁎	0.22⁎	0.38⁎⁎⁎	0.31⁎⁎		
PHQ-9-change	0.34⁎⁎⁎	0.23⁎	0.44⁎⁎⁎	0.24⁎	0.34⁎⁎⁎	0.24⁎	0.07	0.10

Note. PHQ-9: Patient Health Questionnaire-9; Primary Outcome.

WAI-I: Working Alliance Inventory for Guided Internet Intervention; Mediator.

Early-treatment = two weeks after treatment begin, post-treatment = eight weeks after treatment begin. Pre-treatment depression scores were controlled for. The change in depressive symptoms was calculated as the subtract of pre-treatment and post-treatment scores.

⁎ *p* < .05.

⁎⁎ *p* < .01.

⁎⁎⁎ *p* < .001.

### 3.5 Mediation analyses

Overall, the mediation models fit the data with CFI 0.95 or greater, SRMR below 0.08, and RMSEA below or close to 0.06 (Table 4). Thus, the fit of the models tested was acceptable to good.

**Table 4:** Fit-Indices for mediation models.

Mediators	CFI	SRMR	RMSEA
Simple mediation models			
WAI-I (early-treatment)			
Total score	0.96	0.06	0.07
WAI-I Tasks	0.98	0.04	0.05
WAI-I Goals	0.98	0.06	0.04
WAI-I Bond	0.98	0.05	0.04
WAI-I (post-treatment)			
Total Score	0.97	0.06	0.05
WAI-I Tasks	0.99	0.05	0.04
WAI-I Goals	0.99	0.05	0.03
WAI-I Bond	0.99	0.05	0.04
Adherence	0.98	0.04	0.05
Multiple mediation model			
WAI-I early-treatment total score and adherence	0.95	0.06	0.07

Note. WAI-I: Working Alliance Inventory for Guided Internet Intervention.

Adherence: Composite score of clicks, exercises, topics, and time spent on the self-help program. Adherence was calculated for the time between pre- and post-treatment.

#### 3.5.1 Mediation analyses with the total score of working alliance

To test for mediation and assess its effect size, we examined the direct and indirect effect of guidance on depressive symptoms at post-treatment, controlling for the pre-treatment level of depressive symptoms. The results of the mediation analyses are reported in Table 5., For the unstandardized estimates, bootstrapped bias-corrected 95 % confidence intervals were computed. For the WAI-I total scores, the indirect effect differed significantly from zero. Thus, the effect of guidance on depressive symptoms at post-treatment was mediated by the working alliance at early- and at post-treatment. For WAI-I-total at early-treatment, the standardized estimate of the indirect effect was *B* = −0.028, indicating a small effect (accounting for 20.6 % of the total effect). For WAI-I-total at post-treatment, the standardized estimate of the indirect effect was *B* = −0.053, indicating a small to medium effect (accounting for 46.1 % of the total effect).

**Table 5:** Mediation models with total effects, overall direct effects, and overall indirect effects of group assignment (guided/unguided) on post-treatment depressive symptoms.

Mediator	Total effect		Direct effect		Indirect effect	
	Std. Est.	Unstd. Est. [95 % CI]	Std. Est.	Unstd. Est. [95 % CI]	Std. Est.	Unstd. Est. [95 % CI]
Simple mediations						
Early-treatment WAI						
WAI-I total score	−0.136*	−0.150 [−0.309, −0.012]	−0.108	−0.120 [−0.258, 0.020]	−0.028*	−0.031 [−0.092, −0.001]
WAI-I subscales						
Tasks	−0.130*	−0.143 [−0.296, −0.006]	−0.128*	−0.141 [−0.280, −0.005]	−0.002	−0.002 [−0.050, 0.033]
Goals	−0.134*	−0.150 [−0.289, −0.015]	−0.104	−0.115 [−0.248, 0.017]	−0.031*	−0.034 [−0.100, −0.006]
Bond	−0.127*	−0.140 [−0.293, −0.007]	−0.086	−0.094 [−0.251, 0.038]	−0.041	−0.045 [−0.111, 0.002]
WAI-I total score	−0.115	−0.126 [−0.275, 0.006]	−0.062	−0.068 [−0.213, 0.082]	−0.053*	−0.058 [−0.120, −0.017]
Post-treatment WAI						
WAI-I total score	−0.115	−0.126 [−0.275, 0.006]	−0.062	−0.068 [−0.213, 0.082]	−0.053*	−0.058 [−0.120, −0.017]
WAI-I subscales						
Tasks	−0.128*	−0.139 [−0.284, −0.011]	−0.077	−0.083 [−0.224, 0.052]	−0.051*	−0.056 [−0.125, −0.017]
Goals	−0.111	−0.121 [−0.272, 0.017]	−0.059	−0.064 [−0.221, 0.087]	−0.052*	−0.057 [−0.133, −0.012]
Bond	−0.108	−0.119 [−0.269, 0.025]	−0.095	−0.104 [−0.263, 0.054]	−0.014	−0.015 [−0.058, 0.008]
Adherence	−0.119*	−0.131 [−0.276, −0.001]	−0.085	−0.093 [−0.242, 0.044]	−0.034*	−0.037 [−0.077, −0.001]

Multiple mediation						
WAI-I total score early-treatment Adherence WAI-I total score early-treatment and Adherence	−0.130	−0.144 [−0.290, −0.012]	−0.080	−0.089 [−0.229, 0.050]	−0.026 −0.024 −0.050*	−0.029 [−0.086, 0.002] −0.026 [−0.058, 0.005] −0.055 [−0.122, −0.013]

Note. WAI-I: Working Alliance Inventory for Guided Internet Intervention; Adherence: Composite score of clicks, exercises, topics, and time spent on the self-help program. Adherence was calculated for the time between pre- and post-treatment. The significance (*) of the estimates was tested using the bootstrapped bias-corrected 95 % CI. Std. Est. = standardized estimate; Unstd. Est. = unstandardized estimate; CI = confidence interval. The model is corrected for the depression score at pre-treatment. The independent dichotomous variable was group assignment (guided/unguided) and the dependent variable was depressive symptoms (PHQ-9) at post-treatment.

#### 3.5.2 Mediation analyses with the subscales tasks, goals and bond

Of the three subscales of the WAI-I at early-treatment, only the subscale goals mediated the effect of guidance on depressive symptoms. The standardized estimate of the indirect effect was *B =* −0.031, indicating a small mediation effect (accounting for 23.1 % of the total effect). Of the three subscales of the WAI-I at post-treatment, both the subscales tasks (*B* = −0.051; accounting for 39.8 % of the total effect) and goals (*B* = −0.052; accounting for 46.8 % of the total effect) indicated a small to medium mediation effect.

#### 3.5.3 Mediation analysis with adherence

In addition to the models with the working alliance as a mediator, we conducted a simple model with adherence as a mediator. In the simple mediation, adherence significantly mediated the effect of guidance on depressive symptoms. The standardized estimate of the indirect effect was *B* = −0.034, indicating a small mediation effect (accounting for 28.6 % of the total effect).

#### 3.5.4 Multiple mediation analysis with working alliance and alliance

Finally, to test whether adherence and working alliance mediate the effect of guidance irrespective of one another, we conducted a multiple mediation with adherence and early-treatment working alliance as mediators. The standardized estimate of the total indirect effect was *B* = −0.050, indicating a small to medium mediation effect (accounting for 38.5 % of the total effect). Both mediators explained almost equal variance of the total indirect effect (early-treatment working alliance = 52 %, adherence = 48 %).

## 4 Discussion

In this study, we took a closer look at the previous finding that guided participants reported fewer depressive symptoms post-treatment than unguided participants (Bur et al., 2022). We hypothesized that the working alliance plays a role in explaining this finding. Our results support this hypothesis to some extent. First, guided participants reported a higher total working alliance than unguided participants. Second, the working alliance significantly correlated with the change in depressive symptoms for guided and unguided groups (*rs* = 0.16–0.34). Third, the total scores of the working alliance at early- and post-treatment significantly mediated the relationship between guidance and depressive symptoms. Furthermore, the subscale tasks (at post-treatment) and the subscales goals (at early- and post-treatment) mediated the relationship between guidance and depressive symptoms. Fourth, adherence also mediated the relationship between guidance and outcome. Interestingly, when including working alliance and adherence in a multiple mediation model, both variables explained variance of the guidance effect on outcome. Thus, the working alliance seems to contribute to a better outcome independently of adherence.

Compared to unguided participants, guided participants showed a significantly higher working alliance. This difference mainly emerged because guided participants scored significantly higher on the bond subscale. Thus, participants seem to bond more strongly with the treatment providers through additional contact with a psychologist during treatment. At first sight, this finding may not be surprising, but it is especially noteworthy since the alliance was measured quite early in treatment, i.e., two weeks after it began. At this time, guided participants had received just two emails from the treatment providers. Therefore, a small amount of additional contact may be sufficient to strengthen the bond between participants and treatment providers significantly. However, it could also be that not the actual contact itself increases the working alliance; rather that guided participants know a human person will support them during treatment. Therefore, guided participants might perceive the treatment as more credible, more suitable, or have higher treatment expectations (Heim et al., 2018).

A good working alliance seems to be related to a better outcome. The alliance's total score (at early- and post-treatment) significantly correlated with the change in depressive symptoms for the guided group (*rs* = 0.16, 0.34) and for the unguided group (*rs* = 0.26, 0.23). This finding is in line with previous meta-analyses that have found significant correlations (*rs* = 0.20–0.28) between the working alliance and outcomes for guided web-based programs (Flückiger et al., 2018; Kaiser et al., 2021; Probst et al., 2019). However, for depressive symptoms, only half of the studies included in these meta-analyses found a significant association. Therefore, our findings reinforce the notion that the working alliance does play a role in guided web-based programs for depressive symptoms. Furthermore, when looking at the alliance subscales, the subscale tasks was significantly correlated with outcome for guided and unguided conditions at both timepoints, the subscale goals was significantly correlated with outcome for guided and unguided conditions at post-treatment only, and bond was not significantly correlated for guided and unguided conditions. This finding, too, aligns with previous literature for guided web-based programs (Berger, 2017; Gómez Penedo et al., 2020; Probst et al., 2019) and highlights that participants' perception of how well the tasks and goals of a web-based program suits them seems important. Meyer et al. (2015) interpreted a similar finding to mean that participants have a good sense early in the treatment about how helpful an intervention will be. This perceived helpfulness, plausibility, or personal fit might be an essential predictor in internet interventions, whereas the personal bond to the treatment providers might be less critical (Berger et al., 2014). Therefore, treatment providers might attune web-based programs to the preferences and expectations of participants to amplify participants' agreement with tasks and goals of an intervention. Of note, our results suggest that unguided participants might benefit from such an attunement as well.

The working alliance not only correlated positively with change in depressive symptoms but also mediated the relationship between guidance and depressive symptoms (explaining 20.7 % of the total effect at early-treatment and 46.1 % at post-treatment). These findings further highlight the importance of an online working alliance and equal findings from face-to-face studies (Baier et al., 2020). Significant mediations were also found for the subscale tasks (at post-treatment) and the subscale goals (both early- and post-treatment). These findings could be interpreted in line with the term collaboration, which is seen as an essential and cognitive behavioral therapy (CBT)-specific element of the therapeutic relationship (Kazantzis et al., 2017). In CBT, collaboration focuses on the therapist's role as a facilitator of the clients' progress towards his or her own goals. Applied to internet-based self-help, this would mean that the support of a psychologist facilitates this progress as well.

In addition to the working alliance, adherence to the program also significantly mediated the relationship between guidance and depressive symptoms. Thus, it seems that guided participants not only benefit because of a better working alliance with the treatment providers, but also because they engage more with the treatment content than unguided participants. The mediation result of adherence suggests that adherence does not measure something similar to the working alliance, nor does it contradict the working alliance's mediation effect. Rather, both adherence and working alliance individually explain variance of the guidance effect and, consequently, explain more variance together.

We draw two possible practical implications from the results on the relationship between guidance, alliance, and outcome. The first implication is that treatment providers of web-based programs should be made aware of the link between guidance, alliance, and outcome. Treatment providers may assess the working alliance as early as two weeks after treatment begins and intensify or change the mode of support for participants with low early-treatment working alliance (e.g., face-to-face contact).

The second implication of the results may be that the common practice of guiding participants throughout a web-based program could potentially be modified. Although the mediating effect of alliance increases from early- to post-treatment, little contact with treatment providers (two emails in two weeks) at early-treatment already affects working alliance and outcome positively. This could be used as an advantage for internet-based treatments. Instead of guiding participants throughout the entire treatment, it might be equally effective to guide them into the treatment and, possibly, just provide guidance on demand or standardized feedback afterward. While meta-analyses found that guidance is superior to non-guidance (e.g., Karyotaki et al., 2021; Moshe et al., 2021), this does not imply that other forms of guidance and contact are less effective than the guidance usually provided. For example, Zagorscak et al. (2018) found that standardized feedback was equally effective as regular individualized guidance. Furthermore, some studies found no difference in outcomes whether participants were regularly guided or only received guidance on demand (Dahlin et al., 2020; Hadjistavropoulos et al., 2017). Thus, with our early-treatment working alliance findings in mind, it would be interesting to investigate whether initial guidance of two weeks might achieve a similar effect on outcome as guidance throughout treatment. Of note, our study results cannot investigate this hypothesis since participants expected to be guided throughout treatment and not just for two weeks. Therefore, it remains a hypothesis and must be tested in a future study. A reduction of the “dose” of guidance could produce three benefits. First, therapists could spend less time per participant and free up resources. Second, therapists might invest their free resources for participants who do not respond well to treatment and need more guidance. Third, unguided treatments could be significantly improved with little effort, i.e., by adding initial guidance.

### 4.1 Strengths and limitations

To the best of our knowledge, this is the first study to examine alliance as a possible mediator of guidance in a web-based program for depression. Another strength of this study is that the assessment of the early working alliance meets the requirement that a mediator should temporally precede the outcome (Kazdin, 2007). However, this study also has several limitations. First, the general limitations mentioned in the study by Bur et al. (2022) also apply for the analyses presented in this paper (results may not generalize to participants with more severe depressive symptoms, the study sample was self-selected from the community and reliance on self-report measures instead of clinician-administered scales). Second, although the mediation effect of the bond subscale was small to medium, it did not reach statistical significance. This might have been due to too little statistical power. Third, the alliance was measured only twice during treatment. Measuring the alliance repeatedly throughout treatment might reveal more complex relationships between guidance, working alliance and outcome. Such studies might reveal whether the importance of working alliance varies throughout treatment, as has been done for face-to-face psychotherapies (e.g., Volz et al., 2021). Fourth, the study had dropout rates at post-treatment of 31.1 %. A reason for the dropout rate might have been that we have asked participants to use an anonymous email address to ensure privacy. As a result, we may have lost some participants because they did not check this address regularly.

## 5 Conclusion

In this study, guided participants reported a higher total working alliance than unguided participants. The working alliance was significantly correlated with the change in depressive symptoms (*rs* = 0.16–0.34) for guided and unguided participants. The working alliance and adherence to the program both mediated the relationship between guidance and depressive symptoms independently of one another. Furthermore, the participants' agreement on tasks and goals of the web-based program intervention seems to be more important than the bond to treatment providers. Therefore, treatment providers might attune web-based programs to the preferences and expectations of participants. Since working alliance at early-treatment mediates the effect of guidance on outcome, future studies should investigate whether a reduced “dose” of guidance is equally effective as regular guidance.

## Declaration of competing interest

The study was funded by the Department of Clinical Psychology and Psychotherapy of the University of Bern (Switzerland). There was no external funding source. The authors declare that they have no known competing financial interests or personal relationships that could have appeared to influence the work reported in this paper.
